# Exploring molecular links between lymph node invasion and cancer prognosis in human breast cancer

**DOI:** 10.1186/1752-0509-5-S2-S4

**Published:** 2011-12-14

**Authors:** Sangwoo Kim, Hojung Nam, Doheon Lee

**Affiliations:** 1 Department of Bio and Brain Engineering, KAIST, 373-1 Guseong-dong, Yuseong-gu, Daejeon, 305-701, Republic of Korea; 2 Current address: Department of Computer Science and Engineering, University of California at San Diego, 9500 Gilman Dr. La Jolla, CA 92093-0404, USA; 3 Department of Bioengineering, University of California at San Diego, 9500 Gilman Dr. La Jolla, CA 92093, USA

## Abstract

**Background:**

Lymph node invasion is one of the most powerful clinical factors in cancer prognosis. However, molecular level signatures of their correlation are remaining poorly understood. Here, we propose a new approach, monotonically expressed gene analysis (MEGA), to correlate transcriptional patterns of lymph node invasion related genes with clinical outcome of breast cancer patients.

**Results:**

Using MEGA, we scored all genes with their transcriptional patterns over progression levels of lymph node invasion from 278 non-metastatic breast cancer samples. Applied on 65 independent test data, our gene sets of top 20 scores (positive and negative correlations) showed significant associations with prognostic measures such as cancer metastasis, relapse and survival. Our method showed better accuracy than conventional two class comparison methods. We could also find that expression patterns of some genes are strongly associated with stage transition of pathological T and N at specific time. Additionally, some pathways including T-cell immune response and wound healing serum response are expected to be related with cancer progression from pathway enrichment and common motif binding site analyses of the inferred gene sets.

**Conclusions:**

By applying MEGA, we can find possible molecular links between lymph node invasion and cancer prognosis in human breast cancer, supported by evidences of feasible gene expression patterns and significant results of meta-analysis tests.

## Background

The presence of lymph node invasion is one of the strongest indicators for prognoses of distant metastasis and survival in most cancers [1,2]. In the multi-step process of cancer metastasis development, invasion into a vascular or a lymphatic system has generally been believed to be a key step of tumor cell dissemination [3-5]. Once tumor cells acquire abilities of intravasation and survival in an unfavorable vascular environment, they circulate around the whole body parts to form new tumors at the secondary site [6]. While the exact mechanisms of cancer metastasis through blood vessels and lymph nodes are still being studied, it is necessary to explain the processes in a genetic level as a key factor of cancer patients’ prognosis.

Many researchers have devoted their efforts to understand lymph node invasion in breast cancers, because regional lymph nodes are frequently observed as the first site of metastasis [7]. Survival analyses with clinical features showed that lymph node status is generally marked as a top significant factor among conventional clinical features [8-10]. Studies of finding molecular markers using genome-wide expression profiles identified various genetic signatures for prediction of lymph node and distant metastasis [11-19]. However, the associations between conventional clinical features including tumor size, lymph node involvement and distant metastasis (TNM staging [20]) and prognosis are not yet identified in a genetic level. Moreover, the existence of a common gene set for lymph node metastasis in a transcriptional level is unclear [21].

So far, *t*-test based differential expression analysis or clustering methods between lymph node negative and positive samples have been used to detect corresponding gene sets [17-19,21]. Although these methods are straightforward and intuitive, there are several inherent problems in them. First, direct comparison within two classes (lymph node negatives and positives) may simplify the subtle changes over cancer progression. Usually, four-stage pathological N (N0~N3) is used to indicate the degree of lymph node invasion in breast cancer; N0 denotes no lymph node invasion observed. Regarding the expression values as longitudinal data to find patterns over lymph node progression might benefit from utilizing known biomedical information. For instance, a gene whose expression is significantly high only at a certain stage (e.g. N2) is hardly accepted as a closely related gene from current metastasis model. However, a two-class comparison (e.g. N0 vs. others) would mark it differently expressed. Second, the effect of a factor (e.g. lymph node invasion) should be separated from the effect of the others (e.g. tumor size or histological subtype). These factors are generally not independent and will lead to false findings unless carefully analysed. And, the validation of inferred gene signatures should be performed on sufficient number of independent sets in a strict statistical manner. The data statistics and characteristics including inherent biases should be recognized, appropriately treated and be properly analyzed in a meta-analytic way.

There are several statistical models applicable for multivariate correlation scoring (instead of two-class based scoring). Linear/Non-linear (multiple) regression and analysis of variance models (two-way ANOVA and MANOVA) have been widely used in various fields. Both models (linear and non-linear), however, have a few weaknesses; a gene expression pattern over lymph node progression is not necessarily linear, and the data has too few time points to be assessed in a non-linear way. ANOVA models are usually used to test if there is a significant difference among the mean values, so it is not robust to inconsistent fluctuations of expression values. In time series analyses, autoregressive moving average model and its variants (ARMA, ARMAX and ARIMA) are widely used especially in electronic engineering and system identification fields, and some unit root tests (for stationarity test in time series including Augmented Dickey Fuller test [22]) have been used in statistics and econometrics as well. However, there are a few difficulties in adapting these models to our problem; the number of time points is very few, intervals are not regular and the stage is a pseudo-time. After reviewing the conventional models, we developed a new multivariate correlation measure specially designed for non-linear and small data point analysis. Nevertheless, the conventional models were applied as well and tested to compare with our measure and two-class based analyses.

Our method, monotonically expressed gene analysis (MEGA), scores gene expression patterns with their non-linear monotonicity over a stage progression of interest. It accumulates all the normalized expressional differences between two consecutive stages (see Methods). If the direction of expressional change is consistently positive or negative, the score increases; otherwise, the sum of differences will be cancelled out. Because there are two non-independent factors (stage T and N), one variable should be fixed while the other variable is being used. In MEGA, a two dimensional matrix is constructed, each dimension of which is composed of four points (N0~N3 and T1~T4, T0 is excluded due to the lack of data) generating totally 16 data points per a gene. So, applying the scoring function to each row or column represents calculating the cumulative expressional changes over one factor while the other is fixed. MEGA also has a weight parameter to emphasize a specific stage transition (e.g. N1→ N2) to capture genes activated or repressed in a particular time range. After calculating scores, top *k* genes are collected and named N-wise monotonically expressed genes (N-MEG) or T-wise monotonically expressed genes (T-MEG) depending on which factor is used for the analysis. Validation of inferred gene sets can be done in a retrospective way to see how accurately the gene sets classify prognostic outcomes in other independent data. P-values from each test data are integrated by meta-analysis to report more confident accuracy of the gene sets. This is basically one of the most unbiased ways for evaluating usefulness of inferred gene signatures.

If the gene sets show consistence and confident accuracy, a series follow-up analysis can be used for reasoning biological meaning (e.g. common pathways or transcription factors). First, gene set analysis can discover some biological pathways involving in metastasis progression. Considering pathways instead of individual genes as an acting unit of biological phenomena explains how different gene sets are sometimes associated with same conditions. And we can find more succinct way to describe the whole processes. Second, the fact that the genes show similar expression patterns as the cancer metastasis progresses leads us to a hypothesis that some common transcription factors play a crucial role in the process. Here, all the genes are not necessarily causative; rather, they are effect from changes of a fewer number of genes in upper hierarchy. In this case, finding frequently represented motifs from the promoter regions of the gene sets might be a good analysis for discovering the transcription factors. This would be more powerful information in practical applications such as pharmaceutical research and patient treatment.

## Results

Totally four gene sets of size 20 are constructed from 278 breast tumor gene expression data (expO database) by applying monotonically expressed gene analysis (MEGA). They are N-wise monotonically expressed genes (N-MEG) and T-wise monotonically expressed genes (T-MEG), which are further divided into positive and negative correlation sets. Given these four gene sets (N-MEG+, N-MEG-, T-MEG+, and T-MEG-), we tested on65 independent breast cancer prognosis data sets downloaded from ONCOMINE database (See Methods for details) how much the expression values of the genes are correlated with prognostic outcomes.

### Lymph node-wise monotonically expressed genes (N-MEG)

**Figure 1:**
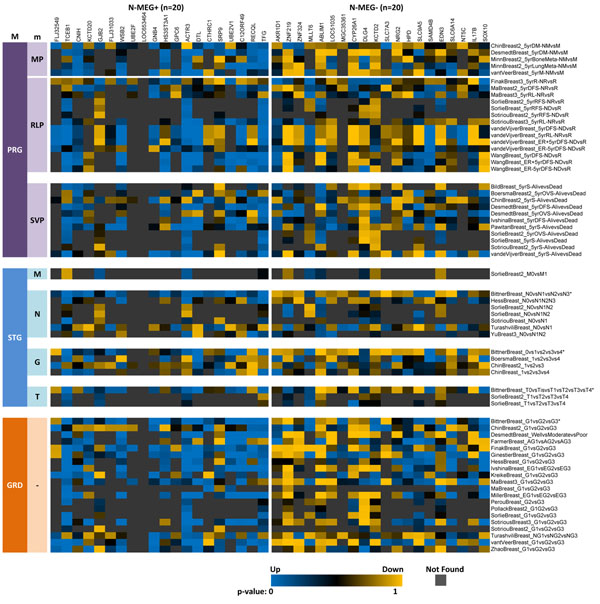
**N-MEG with their meta-analysis test result.** Here, 20 N-MEG+ and 20 N-MEG- genes are tested. Each column corresponds to a specific gene, and each row to an experiment in the ONCOMINE test set. Test set of 65 experiments are classified into three major classes (PRG, STG, and GRD), each of which are subdivided into several minor classes. Blue color denotes up-regulation, and yellow color denotes down-regulation. Experiments with an asterisk (*) denote they used the expO database, and were excluded from further analyses. PRG=prognosis, MP=metastatic prognosis, RLP=relapse prognosis SVP=survival prognosis, STG=stage, M=M stage, N=N stage, G=stage grouping, T=T stage, GRD=tumor grade.

The result of meta-analysis test with N-MEG+ and N-MEG- is shown in Figure 1. Two gene sets are divided by the vertical separator. Three major analysis types (PRG, STG, and GRD) and seven minor analysis types are denoted in the first and second columns. Each row corresponds to an experiment and each column corresponds to a gene. So a value in a cell is a p-value of a gene in the corresponding experiment. Cells are colored blue when the genes are significantly up-regulated at the study, yellow when down-regulated, black when not significantly regulated, and grey when the genes could not be found in the corresponding experiments; here, up-regulation means genes are up-regulated in bad-prognoses, higher stages, and higher grades.

**Figure 2:**
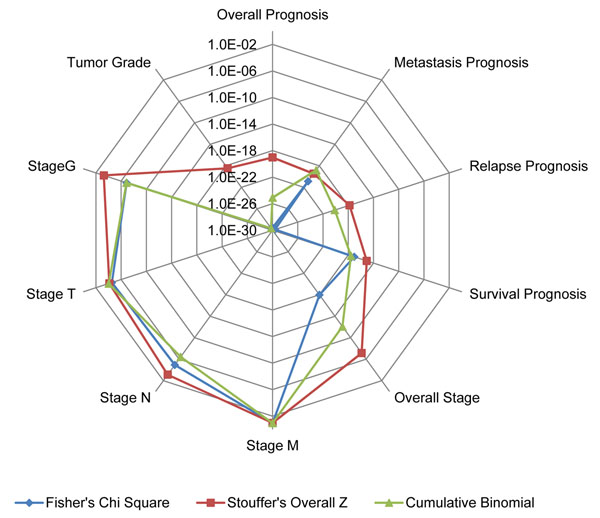
**P-values of N-MEG over ten analysis types.** Each line denotes a different kind of meta-analysis method. Except the ‘stage M’ class, all the p-values are less than 0.01 (and extremely low in prognosis analyses). Each p-value was calculated by multiplying two p-values from 20 up and 20 down genes.

It is easily shown that N-MEG+ genes are positively correlated with worse prognoses, higher tumor stages and higher tumor grades. Similarly, N-MEG- is negatively correlated. From the p-value matrix, we can calculate integrated p-values using three meta-analysis methods over ten test classes (Figure 2). It is easily found that the N-MEG is highly significant in all types of prognosis analyses (p-values less than 10^-14^ in any methods). Except the test for stage M (current status of metastasis), all p-values were less than 0.01. The study of stage M is designed for elucidating differences of gene expression profile between primary tumors and metastatic tumors. The conceptual difference from the prognosis study of metastasis is that while the former describes the status of ‘metastasis occurred’, the latter describes ‘metastasis will occur’. The results of stage N and stage T were intermediately significant; five of the seven studies in the stage N are two class comparisons (N0 vs. others).Correlations with the tumor grade studies were extremely significant. It is also shown that the Stouffer’s Z method gives relatively more conservative results. As the Stouffer’s Z method has been proven to be more robust to a few extreme values [23] and correctable here (see Methods), we will use the corrected version of this method for rest of the study.

**Figure 3:**
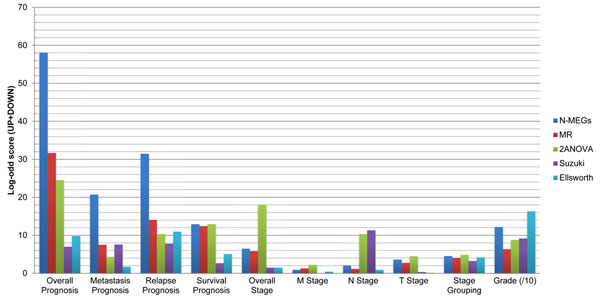
**Comparison with previous studies.** Each value corresponds to a sum of log-odd scores from up and down regulated gene sets. In prognosis analyses, pattern based methods (MEGA, multiple regression and ANOVA models) showed better results than two-class comparison methods (Paired t-test in Suzuki and Mann-Whitney test in Ellsworth). N-MEG (blue) showed the best significance among all the gene sets. Instead, N-MEG and a multiple regression set showed relatively low significance in tumor stage data; probably because most of the N stage test sets used two-class comparison methods. Values in tumor grade analyses were scaled down to 1/10 for better presentation of the graph. N-MEG = N-wise monotonically expressed genes, MR = multiple regression, 2ANOVA = two-way ANOVA.

In a comparison with gene sets from previous work, the N-MEG showed the highest association with cancer prognoses (Figure 3).Gene sets from a multiple regression and a two-way ANOVA model followed it and other two gene sets (Suzuki *et al* and Ellsworth *et al*) showed relatively lower significance. This result implies that the pattern based methods (MEGA, two-way ANOVA and multiple regression models) are more effective than two class direct comparison methods (*t*-test and clustering) in finding prognosis associated genes. On the other hand, ANOVA and Suzuki set showed the best score with the N stage. Like we already mentioned, most of the existing N stage test sets are based on a *t*-test within two classes, which is the same method as what Suzuki *et al* used. In other analysis types including M and T stage grouping and tumor grade, we could not find significant differences among five methods. Abba set was also tested even though the gene set was already had a selection step using prognosis data (selecting 46 top ranked genes from 300 genes, see Methods). The test showed that our gene set was comparable to it (better in metastasis, relapse and overall prognosis) in spite of a significant degree of unfairness.

**Figure 4:**
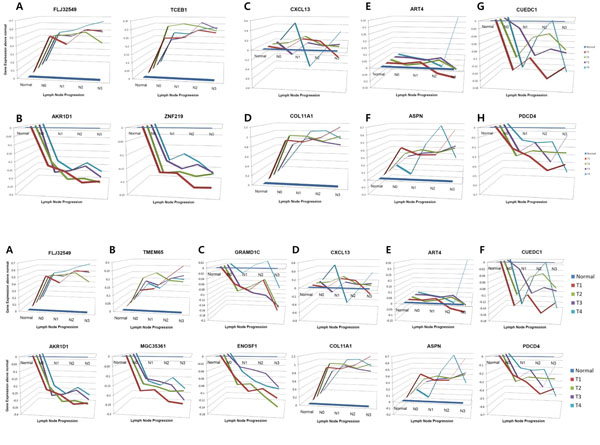
**Aspects of gene expression patterns.** Top ranked genes from four different methods are shown (A: N-MEG, B: multiple regression, C: two-way ANOVA, D: Suzuki set, E: Ellsworth set, and G: Abba set). Genes in the first row are up-regulated genes in lymph node positive samples in each study, the second row are down regulated genes (except in B; ANOVA does not give directional results). Lymph node progression is denoted in X-axis, and the relative expression values against normal breast samples are denoted in Y-axis (in log 2 based fold change). Four different colors are used to discriminate different tumor sizes (T stage 1 to 4). Expression patterns of two genes from N-MEG and multiple regression (FLJ32549, AKR1D1, TMEM65 and MGC35361) show consistent increase (top row) or decrease (bottom row) in all tumor sizes, while top-ranked genes from other studies (D~F) show either inconsistency among different tumor sizes (D up, E and F up) or disagreement over phenotypic traits (D down, E down, and F up). We can see that the multiple regression model find more linear relationships (B) and ANOVA set contains significant fluctuations (C).

Overall aspects of gene expression progression along the N stage give explicit explanations of differences among the candidate gene sets (Figure 4). In the N-MEG and multiple regression model-based gene sets show consistent increase or decrease along the N stage independent to the T stage (Figure 4A and 4B). However, gene sets from two class direct comparison methods (*t*-test and Mann-Whitney test) show certain degree of inconsistency and discrepancy between lymph node phenotypes and gene expression patterns (Figure 4D, 4E, and 4F). This result shows that those gene sets (lymph node positive vs. negative) may contain false positives from abstracting detailed pattern information, and also implicates the reason why N-MEG showed relatively high significance in the prognosis test.

### Classification and survival analysis

**Figure 5:**
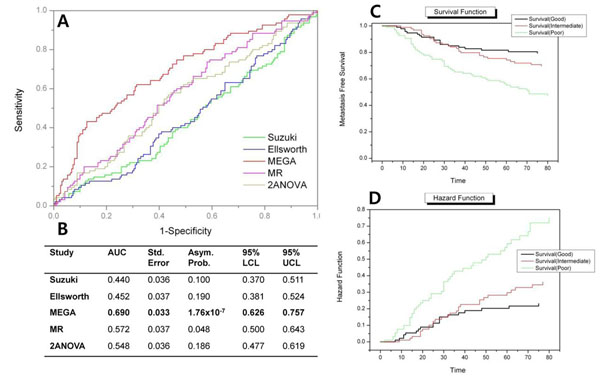
**Classification and survival analysis using N-MEG genes.** (A) An ROC curve shows that the genes from MEGA analysis (N-MEG) have the strongest classification power among other candidate lymph node related genes. (B) Areas under ROC curve (AUC) denote the statistical measurement of the classification power. Note that the genes from previous two-class comparisons hardly prove their usefulness in classification; but the result may differ when conducted in other classification methods. (C and D) Three risk groups (good, intermediate and poor) show distinct survival and hazard functions in Kaplan-Meyer diagrams. AUC=area under curve, Std. Error=standard error, Asym. Prob.=Asymptotic probability, LCL=lower confidence limit, UCL=upper confidence limit.

To show the classification power of the N-MEGs, we conducted a test for 5-year metastasis free survival data from Wang et al [15]. Because the meaning of N-MEG+ and N-MEG- is so clear, we scored the sum of row-normalized z-scores of corresponding genes; adding for 20 N-MEG+ genes and subtracting for 20 N-MEG- genes. For the 286 primary breast samples (91 metastasis in 5-years), the mean score was nearly zero (6.5x10^-13^) and the standard deviation was 7.8. From the 51 patients whose scores were bigger than the mean plus one standard deviation, 37 had metastasis in 5-years giving 0.35 of sensitivity and 0.92 of specificity. The overall accuracy was 0.71. An ROC curve was drawn to compare the N-MEGs with other gene sets (Figure 5A). The N-MEGs showed the best classification power. Interestingly, while the two statistical approaches using stage progression (multiple regression and two-way ANOVA) managed to prove a certain degree of usefulness, the studies using two class comparisons did not. Although the result may be further improved by other fancy classifiers with optimization procedures, we can tentatively conclude that observing the signatures of stage progression gives better results. A set of area under curve (AUC) were denoted in Figure 5B. The AUC of MEGA was 0.69 (0.626 ~ 0.757 in 95% confidence limits). To conduct a survival analysis we divided the all 286 patients into three groups of equal size (n=95 for good and poor group, 96 for intermediate group). It is shown that the three groups have distinct metastasis free survival and hazard rate in Kaplan Meier estimation (Figure 5 C and D).

### T-wise monotonically expressed genes (T-MEG) and comparison with N-MEG

T-MEG (n=40, 20 T-MEG+ and 20 T-MEG-) were also significantly correlated with breast cancer prognosis including metastasis and relapse, but the significance was generally worse than N-MEG (Table 1). In the prognosis of metastasis studies, both of the T-MEG+ and T-MEG- were significant (p-values of 4.3x10^-8^ and3.1x10^-3^respectively), but they were not as effective as N-MEG (p-values of 1.2x10^-15^ and1.4x10^-6^). This result agrees with the previously known pathological facts; both of the degree of lymph node invasion and tumor size are important in predicting metastasis probabilities, while the former gives more direct evidences. We can also notice that tumor size related gene were either not significant (in prognosis of survival and tumor stages) or less significant than lymph node invasion related genes (in prognosis of relapse and tumor grade).

The distinct characteristics between the two tumoral features might be tumor tissue specific. Breasts are not essential organs for personal survival. So even though a tumor has grown to be large, the cancer is not a fatal disease unless the tumor has been spread to other organs. In this case, mastectomy would be effective for improving patients’ survival rate. Although cancer prognosis is a result of complex and stochastic activities among cellular processes, we can conclude this tendency would be valid for other non-essential organs.

**Table 1:** Comparison of N-MEG and T-MEG in a meta-analysis test

**Analysis**	**N-MEG (n=40)**		**T-MEG (n=40)**	
	
	**N-MEG+ (n=20)**	**N-MEG- (n=20)**	**T-MEG+ (n=20)**	**T-MEG- (n=20)**
**Metastasis Prognosis**	1.2x10^-15^	1.4x10^-6^	4.3x10^-8^	3.1x10^-3^
**Relapse Prognosis**	7.9x10^-27^	7.8x10^-6^	7.6x10^-4^	2.1x10^-7^
**Survival Prognosis**	5.7x10^-13^	0.21	0.41	0.013
**Overall Prognosis**	9.4x10^-51^	1.9x10^-8^	1.5x10^-6^	1.1x10^-9^
**Stage M**	0.38	0.28	0.91	2.8x10^-4^
**Stage N**	0.017	0.10	0.15	0.57
**Stage T**	2.1x10^-3^	0.074	0.098	0.15
**Stage G**	0.063	7.2x10^-3^	0.93	0.18
**Overall Stage**	2.1x10^-4^	1.2x10^-3^	0.46	0.075
**Tumor Grade**	1.3x10^-104^	1.3x10^-8^	2.3x10^-8^	6.0x10^-10^

N-MEG showed better significance than T-MEG. Generally speaking, N-wise progression gives more information than T-wise progression in cancer prognosis.

### Genes related to specific N stage transitions

We further tested the significance of genes related to specific steps of lymph node invasion progression by altering a leaping factor β. The leaping factor was set to 10 and applied to three different steps (N0→N1, N1→N2, and N2→N3) with α factor remains to be zero. These gene sets are N-wise monotonically expressed genes with a leaping at a specific stage transition A→B (N-MEG^A→B^: here, N-MEG^0→1^, N-MEG^1→2^, and N-MEG^2→3^ respectively). Interestingly, we found significant discrepancy among lymph node invasion progression steps (Table 2). Genes which were significantly up or down regulated in the N1→N2 progression (N-MEG^1→2^) were of no significance in most of the prognosis and tumor stage studies (p-values > 0.01). Instead, N-MEG^0→1^ and N-MEG^2→3^ were significant in most of the studies including prognosis of metastasis, prognosis of relapse, prognosis of survival and tumor grade.

**Table 2:** Comparison of stage transition specific genes

**Analysis**	**N-MEG^0^**^→^**^1^ (n=40)**		**N-MEG^1^**^→^**^2^ (n=40)**		**N-MEG^2^**^→^**^3^ (n=40)**	
	
	**+**	**-**	**+**	**-**	**+**	**-**
**MP**	2.8×10^-4^	0.13	0.24	0.13	5.7×10^-12^	3.8×10^-11^
**RLP**	7.7×10^-7^	1.0×10^-15^	0.13	0.084	4.8×10^-11^	2.3×10^-6^
**SVP**	5.9×10^-4^	0.03	0.75	0.53	1.2×10^-10^	1.4×10^-5^
**OVP**	1.2×10^-11^	9.3×10^-13^	0.24	0.08	7.8×10^-29^	2.0×10^-17^
**STM**	0.042	0.14	0.97	0.30	0.043	0.035
**STN**	0.030	3.5×10^-6^	0.04	0.068	9.4×10^-4^	0.06
**STT**	1.8×10^-3^	2.4×10^-10^	0.12	0.069	5.9×10^-7^	0.75
**STG**	1.1×10^-5^	6.9×10^-9^	0.01	0.015	0.11	0.53
**Overall Stage**	6.6×10^-8^	2.3×10^-20^	4.2×10^-3^	1.4×10^-3^	7.0×10^-8^	0.15
**Tumor Grade**	2.1×10^-16^	2.9×10^-22^	7.2×10^-6^	0.48	1.1×10^-42^	1.7×10^-58^

N-MEG^2→3^ (β=10) showed the best significance in cancer prognosis test sets. While N-MEG^0→1^was also significantly associated with prognosis and some stages, N-MEG^1→2^ showed surprisingly insignificant results. MP=prognosis of metastasis, RLP=prognosis of relapse, SVP=prognosis of survival, OVP=overall prognosis, STM=stageM, STN=stageN, STG=stageG.

Firstly, we expected that N-MEG^0→1^ would be more informative than N-MEG in the other stage transitions. Because, it is thought that if a set of tumor cells acquire high motility to migrate and intravasate into lymph nodes, dissemination of tumor cells over the larger parts of lymph nodes would follow spontaneously [5]. But the result of meta-analysis test represents that there would be another transcriptional changing event in the late step of lymph node invasion before raising distant metastasis.

To inspect the characteristics of N-MEG^0→1^ and N-MEG^2→3^, we chose 200 genes from each gene set (100 positive and 100 negative genes in N-MEG^0→1^ and N-MEG^2→3^) and compared them each other. We found that there were few overlaps between two gene sets; no overlap in top 20 genes, and only two overlaps in 200 genes. But in the gene function analysis using Gorilla [24], both gene sets were enriched in the immune response GO terms (p-values ~ 1.0×10^-4^). Where the immune response is a well-known process affecting lymph node invasion [25-27], it is convincing that both gene sets are distinct but closely related to lymph node invasion by connected pathways (see Additional Files 1 and 2 for full enrichment map).

### Pathway analysis of N-wise progression

To observe changes of pathways in N-wise progression, we applied Gene Set Enrichment Analysis (GSEA) [28] to N-MEG. All 20,073 genes were sorted by their LE’ scores in descendant order. And the sorted list was analyzed by the GSEA Preranked test using 1,186 curated MSIGDB gene sets (C2-CGP: chemical and genetic perturbations). In the N-MEG+ genes, we found that top ranked enriched pathways were closely related to T cell activities, cell differentiation and wound healing pathways (Table 3). It is previously known that immune response has dual role in tumor initiation and progression (reviewed in [26]) – inhibition of tumor growth by antitumor cytotoxic T-cell activities (reviewed in [29]), and promotion of tumorigenesis, invasion and metastasis by arising chronic inflammatory environment [30-32]. Recently, DeNardo *et al* found that CD4+ T cell promotes lung metastasis of breast cancer through macrophages [33]. These evidences are in concordance with our N-MEG+ result by supporting that maintenance or increase of N-wise gene expression is closely correlated with lymph node invasion and poor prognosis. It is also well known that a serum response of fibroblasts including wound healing pathways efficiently predicts cancer progression [34]. Other two gene sets in the Table 3 are from previously studied result about general differentiation of tumor cells (tumor grade) and prognosis, which support that the N-MEG+ are negatively correlated with differentiation and prognosis.

**Table 3:** Enriched pathway analysis of N-MEG+ using GSEA

R	Gene Set Name	Size	ES	NES	P	FD	Gene Set Description
1	WIELAND_HEPATITIS_B_INDUCED*	96	0.62	3.10	0	0	Up-regulated with adaptive T cell activities in viral clearance
2	LEE_TCELLS3_UP*	103	0.63	3.05	0	0	Up-regulated in immature T cell in CD4+ T cell differentiation
3	CANCER_UNDIFF_META_UP^@^	69	0.65	3.05	0	0	Up-regulated in undifferentiated tumor cells
4	SERUM_FIBROBLAST_CELLCYCLE^&^	137	0.59	3.02	0	0	Up-regulated in serum response of fibroblasts (wound healing)
5	BRCA_PROGNOSIS_NEG^@^	95	0.62	2.98	0	0	Up-regulated in breast tumor cells of negative results (prognosis)

Top five highly enriched pathways are shown. * T cell mediated immune response pathways, ^&^ Serum response related pathway, ^@^ Gene sets previously known as bad prognosis. R=rank, P=nominal p-value, FD=FDR q-value.

### Common TF binding site prediction for N-MEG

**Figure 6:**
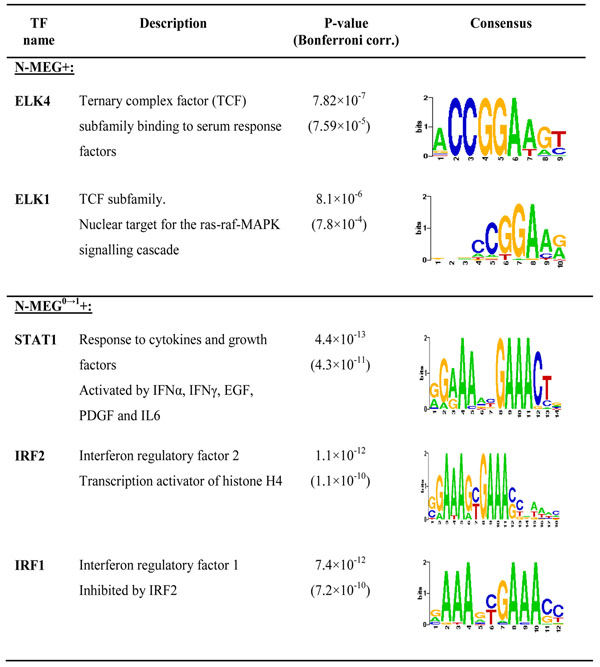
**Candidate commonly binding transcription factors.** Using Pscan, 50 top-ranked N-MEG+ and N-MEG^0→1^+ were analysed in their upstream sequences. In N-MEG+, transcription factors involved in serum response activities have been identified. In N-MEG^0→1^+, IRF1-IRF2 mediated tumor suppressing pathways were identified as candidate driving pathways.

Because the N-MEG+ and N-MEG- are already selected from their expression patterns along the lymph node invasion progression, we can hypothesize that they are co-regulated by several core transcription factors. To find candidate common transcriptional regulators, we analyzed upstream regions of the N-MEG and selected significantly over-represented motif binding sites using Pscan program [35]. We selected top 20, 30, and 50 N-MEG and obtained matching mRNA RefSeq sequences from DAVID database [36,37]. By running Pscan on [-450, +50] upstream regions onto the JASPAR database [38], we found that ELK4 and ELK1 binding sites were significantly over-represented (p-values 7.8×10^-7^ and 8.1×10^-6^ respectively, data shown in Figure 6). ELK4 and ELK1 (E26 Like Transcription Factor) are previously known as members of ternary complex factor (TCF) subfamily, which forms a ternary complex by binding to the serum response factor and the serum response element in a promoter region of the c-fos proto-oncogene [39] (SAP1 is a previous name for ELK4). This finding supports the results of the pathway analyses in the previous section implicating that ELK4 based serum response mechanism might be a driving force for breast cancer lymph node invasion and metastasis.

We also conducted the same analysis with N-MEG^0→1^ and N-MEG^2→3^ (β=10). While there was no significant common binding sites in N2→N3 progression, we found STAT1, IRF2, and IRF1 can be common binding transcription factors in 50 N-MEG^0→1^ (p-values of 4.4×10^-13^,1.1×10^-12^, and7.4×10^-12^ respectively, data shown in Figure 6). We found that IRF1, who plays a tumor suppressing role, is negatively regulated by competitive transcriptional binding of IRF2, both of which were significantly correlated with tumor stage (p-value 0.001), depth of tumor infiltration (p-value 0.006) and lymph node metastasis (p-value 0.015) in human esophageal cancers [40]. Here, we also suggest that IRF2-IRF1 pathway is likely to be involved in lymph node invasion and metastasis progression in human breast cancers with well-known activities of STAT1 [41-44].

## Discussion

In this study, we proposed a monotonically expressed gene analysis (MEGA) for extracting genes that are related to lymph node invasion and tumor size in breast cancer. We analysed expression patterns over a two dimensional N×T space and provided results of meta-analysis to evaluate the gene sets. The test has been conducted on completely independent data sets. We showed that gene sets selected from the suggested LE’ and TE’ functions are strongly correlated with cancer prognoses including metastasis, relapse and survival, and showed significantly better results than conventional approaches. These functions are specially designed to capture expressional differences between two consecutive stages and consistency of expression patterns as well. The MEGA model also enabled us to analyze the impact of each clinical factor independently, and to inspect a specific stage transition in a cancer progression.

**Figure 7:**
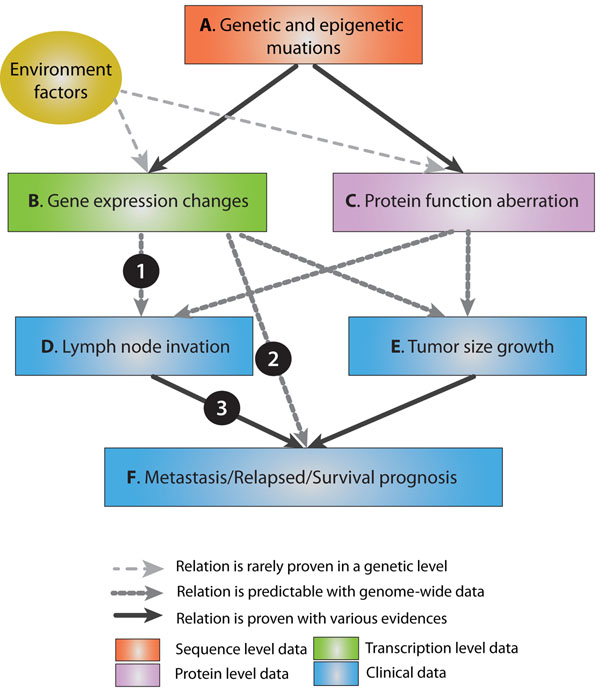
**Conceptual relationship of clinical and genetic factors.** Colors in the box denote different levels of data. Lines are drawn in different shapes according to different degrees of certainty. After primary tumor occurred, accumulated (epi)genetic mutations drive cancer progression. With transcriptional level data, we can elucidate lymph node invasion (A) and cancer prognosis (C). But in the prediction steps, the previously proven relation between D and F should be properly explained (B).

Before concluding our report, it is necessary to reconsider the meaning of linking clinical factors and cancer prognosis in a molecular level. A general relationship among clinical and genetic factors is described in Figure 7. Since a primary tumor first occurred, accumulated genetic and epigenetic aberrations drive the tumor’s progression. The relationship between genetic aberrations and gene expression changes or protein function aberration is strongly established. So it is obvious that progressed tumors have different gene expressions or abnormal proteins. In our MEGA analysis, we focused on the gene expression part. If we found a candidate gene set which connects gene expression changes and lymph node invasion (A in Figure 7), it should be able to explain the relationship to cancer prognosis (C in Figure 7), because the correlation between lymph node invasion and cancer prognosis has been firmly proven (B in Figure 7). We found that previous candidate genes in A rarely found proper explanations of C. Here, the meaning of our study is summarized in two points. First, we tried to improve accuracy in finding candidate genes in A by interpreting gene expression patterns over lymph node progression (MEGA). Second, we provided a credible meta-analysis test procedure to validate the relationship in C. As there still unexplained important factors remain, we have to integrate additional data of other levels to finalize the lymph node invasion related (or causing) genes. But we suggest the future work also should be cross-validated in the different types of relations.

Although the MEGA analysis provided a feasible link to clinical factors and cancer prognoses in a genetic level, some parts remain to be improved. First, the monotonicity can be defined in various ways. Currently we can rarely determine the activities of genes in an absolute expression level. Some genes have higher saturation level so that the gene expression pattern might show a monotonic increase through all the clinical stages. On the other hand, if an activity of a gene is easily saturated, the gene expression pattern above a certain degree would not be informative anymore. Handling and determining the optimized pattern on every gene is almost impossible, but other heuristics will be available using kinetics and text-mining. Second, integration with other omics data always count. It is still a big question; how to connect two different types and levels of information. As shown in Figure 7, protein level information explains another big part of clinical outcomes. In our opinion, those information sets should be integrated to an augmented ‘gene’ entity with other available information like point mutations, SNPs, and CNVs. In this case, the MEGA model has to be revised in its scoring functions. Lastly, finding for driving mechanisms of progression is one of the ultimate goals in this field. We tried to elucidate these mechanisms through pathway analyses and commonly binding transcription factor analyses here, but it is yet to be a striking discovery. After we solve the prior questions, our approach might be more helpful in clarifying the core genes or genetic events that can be essential in therapeutic applications.

## Methods

### Data sets

#### Training data sets

We used 278 breast tumor gene expression data from the expO (expression project for Oncology) database (http://www.intgen.org/expo.cfm, International Genomics Consortium). The data can be also downloaded from NCBI’s GEO database (GSE2109). From 2,158 gene expression profiles for all tissues and tumor types, we chose only breast carcinomas. Samples without pathological N and T stage records, or whose pathological M stage and histological information indicate inclusion of distant metastasis were removed. Finally, the 278 non-metastasis breast tumor samples were categorized into 16 N×T classes (N0~N3, T1~T4). We also used seven normal breast gene expression profiles from GSE3744 [45] to infer the deviation of each N×T stage against the normal condition. Normalization of data was processed with the Simpleaffy Package [46] in R by applying RMA normalization for every N×T stage with normal breast samples. After normalization, probe sets were collapsed into gene symbols using the GSEA collapse tool [28]. Each gene was scored by log 2 based fold change.

#### ONCOMINE data sets for test

For test sets, we used 65 breast cancer data sets from ONCOMINE database [47]. The 65 data sets were firstly classified into three major analysis types (Prognosis, Tumor Stage, and Tumor Grade) each of which is further classified into matching minor subtypes. Minor typing has been done by manual inspection. From the ONCOMINE database, we could download pre-analyzed tables which include sample size, statistics, and two-tailed p-values. Two-tailed p-values were further converted into one-tailed p-values.

### Monotonically expressed gene analysis (MEGA)

In this work, we use two clinical variables (pathological T and N), but MEGA can be expanded to three or more variables with similar procedures. The final goal of the MEGA analysis is to extract N-wise monotonically expressed genes (N-MEG) and T-wise monotonically expressed genes (T-MEG) using monotonicity functions. We first define a two dimensional N×T space for each gene. The N×T space for a gene g_y_ consists of p numbers of N stages and q numbers of T stages can be defined as a (p+1)×(q+1) matrix X:

where the first row and the first column denote gene expressions of normal samples.

To represent how consistently a series of gene expressions has changed along N and T axes, we defined two scoring functions of the X matrix:

where LE is a monotonicity function of gene expression over lymph node invasion progression and TE is monotonicity function of gene expression over tumor size growth. The parameter α is a consistency factor which emphasizes the direction of gene expression changes, S is a sign function, and β is a leaping factor for giving weights to an specific step of stage progression. The characteristics of parameter α were not explored in this study. The sign function S is defined as:

And a matrix of leaping factors β is defined as a matrix form:

Because β is only meaningful in two consecutive stages, the **β** matrix is much like a diagonal matrix. The off-diagonal entries are always defined as 1. In this study, we applied the leaping factors only for N stage progression.

We can interpret the value of LE and TE functions as a sum of moving deviations in the course of N and T stage progression. For each stage, we calculate the gene expression differences between the current stage and previous stages from the beginning of stage to the one step before the current stage. If a certain gene shows a monotonic increase or decrease in its gene expression along the N or T stages, the absolute value of the function would be larger.

Finally the LE and TE functions are normalized by the overall standard deviation of the **X** matrix:

We selected top 40 genes for each monotonicity function (LE’ and TE’) with their absolute scores. The 40 genes are composed of 20 genes of high score and 20 genes of low score. The genes of high LE’ score means that the expression of those genes showed monotonic increase as the lymph node invasion progresses, so the set of the genes is named N-wise monotonically expressed genes with positive correlation (N-MEG+). Similarly, N-wise monotonically expressed genes with negative correlation (N-MEG-) and two other gene sets on a tumor size factor (T-MEG+ and T-MEG-) were defined.

### Meta-analysis test on ONCOMINE data set

We performed meta-analysis tests on 65 ONCOMINE data sets with the selected N-MEG and T-MEG. Assume that we have a gene set G = {g_1_, g_2_…g_n_} and an experiment set E = {e_1_, e_2_…e_k_}. For a gene i and experiment j, we can extract a p-value of gene i in the experiment j from the ONCOMINE data set:

Because the p_ij_ is basically two tailed p-value in the original data sets, we converted these p-values into one-tailed p-values:

During this procedure, some genes were missed due to the different naming strategies and the difference of coverage among the test sets. To minimize the loss of information, we searched for all aliases and symbols of earlier versions using the recent version of HUGO Gene Nomenclature Committee (HGNC) database (2009/08/23).

Three meta-analysis methods have been applied to calculate overall p-values for a certain set of experiments – Fisher’s inverse chi-square [48], Stouffer’s overall Z [49], and cumulative binomial distribution. Here, the Fisher’s inverse chi-square method computes a combined statistic using,

which follows a χ^2^distribution with 2nk degrees of freedom under the joint null hypothesis [50]. Unweighted Stouffer’s Z was calculated by transforming every p-value into z-score upon the standard normal distribution, followed by summing up all z values and dividing by square root of the total numbers:

Here, the  is a standard normal distribution, Z is the sum of all z-values. For the cumulative binomial method, we first set a threshold to determine whether a given p-value is significant or not. From the total nk numbers of p-values, we count the significant p-value number n_s_. For given a threshold p_h_, a probability that one can get a number of p-values equal to or more than n_s_ incidentally is,

which can be approximated using incomplete beta function.

So, the final p-value is calculated like below.

### Correction of Z scores from background biases

Before finalizing p-values of meta-analyses, we noticed that the p-values in the ONCOMINE data sets are upwardly biased. In the 65 studies, 55 studies have bigger number of significantly changed genes than we expected. And we also found that 43 studies have more up-regulated genes than down-regulated genes. We do not insist that these results mean experimental errors; we would rather think it is natural that many of genes are going to be actively expressed as the cancer progresses and regulatory mechanisms are being broke down. But in the case of test procedures, we are likely to get more false positives unless we consider the background biases. For example, some random gene sets may represent ‘more than average’ results and will be thought to be significant for cancer phenotypes.

To correct the background biases, we generated 1,000 random gene sets (n=20) and tested on the ONCOMINE data sets. And we computed Stouffer’s overall Z score on each random gene set. Averaged Z scores represent an expected Z score from a random gene set. From this result, we could conclude that the meta-analysis result of gene sets look more up-regulated in the cancer progression than they really are. So we corrected all the Stouffer’s Z scores result from N-MEG and T-MEG by subtracting the mean values of Z in the up-regulation test and adding in the down-regulation test.

### Comparing with previous studies

We first extracted lymph node invasion related gene sets from previous studies (Suzuki *et al *[17], Abba *et al *[18], and Ellsworth *et al *[19]). Each gene set was tested using the corrected Stouffer’s Z test described in previous section. We found that the gene set from Abba *et al* was already reduced from 300 to 46 genes using eight prognosis experiments. So the Abba set was not used in further comparisons. We also found there are four experiments which used expO data set in the ONCOMINE test set (Bittner *et al*). All the overlapping data was excluded in the test procedure. Additionally, we selected 40 genes from expO data using two-way ANOVA and multiple regression models. For each gene, both models were constructed using ‘aov’ and ‘lm’ functions in R. In ANOVA models, genes were sorted by N stage dependent two-tailed p-values derived from their F-statistics because of the model’s non-directionality. In multiple regression models, 40 genes with the highest P-values of N stage were selected where their T stage and interaction terms are not significant. Directions of regulation were determined from the estimated coefficients (up >0, down <0). Finally five gene sets (N-MEG, multiple regression set, two-way ANOVA set, Suzuki set, and Ellsworth set) were tested and their p-values were reported. P-values from meta-analysis were converted into log-odd score using –log_10_(P). For each study, the final score was calculated from adding the two log-odd scores (from up and down regulated gene sets).

## Competing interests

The authors declare that they have no competing interests.

## Authors' contributions

SK and DL designed the study. SK and HN performed the experiments. All authors helped draft and edit the final manuscript.

## Supplementary Material

Additional File 1 **Analysis of enriched GO Term with GOrilla (N-MEG^0→1^).** Genes related to N0→N1 stage transition showed significant over-representation with collagen and extracellular matrix constituent. Click here for file

Additional File 2 **Analysis of enriched GO Term with GOrilla (N-MEG^2→3^).** Genes related to N2→N3 stage transition showed significant over-representation with immune response, wound response and inflammation. Click here for file

## References

[B1] McGuire WL Prognostic factors for recurrence and survival in human breast cancer Breast Cancer Research and Treatment 1987 10 5 9 10.1007/BF01806129 3689982

[B2] Foster RS Jr The biologic and clinical significance of lymphatic metastases in breast cancer Surgical oncology clinics of North America 1996 5 79 8789495

[B3] Christine LC Carol A Donald EH Relation of tumor size, lymph node status, and survival in 24,740 breast cancer cases Cancer 1989 63 181 187 10.1002/1097-0142(19890101)63:1<181::AID-CNCR2820630129>3.0.CO;2-H 2910416

[B4] Fidler IJ THE PATHOGENESIS OF CANCER METASTASIS: THE 'SEED AND SOIL' HYPOTHESIS REVISITED Nature Reviews Cancer 2003 3 453 458 10.1038/nrc1098 12778135

[B5] Gupta GP Massague J Cancer Metastasis: Building a Framework Cell 2006 127 679 695 10.1016/j.cell.2006.11.001 17110329

[B6] Nguyen DX Massague J Genetic determinants of cancer metastasis Nat Rev Genet 2007 8 341 352 17440531 10.1038/nrg2101

[B7] Stacker SA Achen MG Jussila L Baldwin ME Alitalo K Metastasis: Lymphangiogenesis and cancer metastasis Nat Rev Cancer 2002 2 573 583 10.1038/nrc863 12154350

[B8] Jatoi I Hilsenbeck SG Clark GM Osborne CK Significance of Axillary Lymph Node Metastasis in Primary Breast Cancer J Clin Oncol 1999 17 2334 2340 10561295 10.1200/JCO.1999.17.8.2334

[B9] Nasser IA Lee AK Bosari S Saganich R Heatley G Silverman ML Occult axillary lymph node metastases in node-negative breast carcinoma Human pathology 1993 24 950 10.1016/0046-8177(93)90108-S 7504652

[B10] Gasparini G Weidner N Bevilacqua P Maluta S Dalla Palma P Caffo O Barbareschi M Boracchi P Marubini E Pozza F Tumor microvessel density, p53 expression, tumor size, and peritumoral lymphatic vessel invasion are relevant prognostic markers in node- negative breast carcinoma J Clin Oncol 1994 12 454 466 7509851 10.1200/JCO.1994.12.3.454

[B11] Sorlie T Gene expression patterns of breast carcinomas distinguish tumor subclasses with clinical implications Proc Natl Acad Sci USA 2001 98 10869 10874 10.1073/pnas.191367098 11553815 PMC58566

[B12] van de Vijver MJ A gene-expression signature as a predictor of survival in breast cancer N Engl J Med 2002 347 1999 2009 10.1056/NEJMoa021967 12490681

[B13] van/'t Veer LJ Gene expression profiling predicts clinical outcome of breast cancer Nature 2002 415 530 536 10.1038/415530a 11823860

[B14] Ramaswamy S Ross KN Lander ES Golub TR A molecular signature of metastasis in primary solid tumors Nature Genet 2003 33 49 54 10.1038/ng1060 12469122

[B15] Wang Y Klijn JGM Zhang Y Sieuwerts AM Look MP Yang F Talantov D Timmermans M Meijer-van Gelder ME Yu J Gene-expression profiles to predict distant metastasis of lymph-node-negative primary breast cancer The Lancet 2005 365 671 679 10.1016/S0140-6736(05)17947-1 15721472

[B16] Sun Y Goodison S Li J Liu L Farmerie W Improved breast cancer prognosis through the combination of clinical and genetic markers Bioinformatics 2007 23 30 37 10.1093/bioinformatics/btl543 17130137 PMC3431620

[B17] Suzuki M Tarin D Gene expression profiling of human lymph node metastases and matched primary breast carcinomas: clinical implications Molecular oncology 2007 1 172 180 10.1016/j.molonc.2007.03.005 19383293 PMC5543892

[B18] Abba MC Sun H Hawkins KA Drake JA Hu Y Nunez MI Gaddis S Shi T Horvath S Sahin A Breast cancer molecular signatures as determined by SAGE: correlation with lymph node status Molecular Cancer Research 2007 5 881 10.1158/1541-7786.MCR-07-0055 17855657 PMC4186709

[B19] Ellsworth RE Seebach J Field LA Heckman C Kane J Hooke JA Love B Shriver CD A gene expression signature that defines breast cancer metastases Clinical and Experimental Metastasis 2009 26 205 213 10.1007/s10585-008-9232-9 19112599

[B20] Singletary SE Connolly JL Breast Cancer Staging: Working With the Sixth Edition of the AJCC Cancer Staging Manual CA Cancer J Clin 2006 56 37 47 10.3322/canjclin.56.1.37 16449185

[B21] Weigelt B Wessels LFA Bosma AJ Glas AM Nuyten DSA He YD Dai H Peterse JL van't Veer LJ No common denominator for breast cancer lymph node metastasis Br J Cancer 2005 93 924 932 10.1038/sj.bjc.6602794 16189523 PMC2361648

[B22] Dickey DA Fuller WA Distribution of the estimators for autoregressive time series with a unit root Journal of the American statistical association 1979 427 431

[B23] W MC Combining probability from independent tests: the weighted *Z*-method is superior to Fisher's approach Journal of Evolutionary Biology 2005 18 1368 1373 10.1111/j.1420-9101.2005.00917.x 16135132

[B24] Eden E Navon R Steinfeld I Lipson D Yakhini Z GOrilla: a tool for discovery and visualization of enriched GO terms in ranked gene lists BMC Bioinformatics 2009 10 48 10.1186/1471-2105-10-48 19192299 PMC2644678

[B25] Noriaki S Hiroyuki K Masayuki W Soichiro M Masaki M Keizo S Local immune response to tumor invasion in esophageal squamous cell carcinoma: The expression of human leukocyte antigen-DR and lymphocyte infiltration Cancer 1994 74 586 591 10.1002/1097-0142(19940715)74:2<586::AID-CNCR2820740209>3.0.CO;2-4 8033037

[B26] de Visser KE Eichten A Coussens LM Paradoxical roles of the immune system during cancer development Nat Rev Cancer 2006 6 24 37 10.1038/nrc1782 16397525

[B27] Byrne KJO Dalgleish AG Browning MJ Steward WP Harris AL The relationship between angiogenesis and the immune response in carcinogenesis and the progression of malignant disease European journal of cancer (Oxford, England : 1990) 2000 36 151 169 10.1016/S0959-8049(99)00241-5 10741273

[B28] Subramanian A Tamayo P Mootha VK Mukherjee S Ebert BL Gillette MA Paulovich A Pomeroy SL Golub TR Lander ES Mesirov JP Gene set enrichment analysis: A knowledge-based approach for interpreting genome-wide expression profiles Proceedings of the National Academy of Sciences of the United States of America 2005 102 15545 15550 10.1073/pnas.0506580102 16199517 PMC1239896

[B29] Zou W Immunosuppressive networks in the tumour environment and their therapeutic relevance Nat Rev Cancer 2005 5 263 274 10.1038/nrc1586 15776005

[B30] Leek RD Lewis CE Whitehouse R Greenall M Clarke J Harris AL Association of Macrophage Infiltration with Angiogenesis and Prognosis in Invasive Breast Carcinoma Cancer Res 1996 56 4625 4629 8840975

[B31] Lin EY Pollard JW Tumor-Associated Macrophages Press the Angiogenic Switch in Breast Cancer Cancer Res 2007 67 5064 5066 10.1158/0008-5472.CAN-07-0912 17545580

[B32] Pollard JW Tumour-educated macrophages promote tumour progression and metastasis Nat Rev Cancer 2004 4 71 78 10.1038/nrc1256 14708027

[B33] DeNardo DG Barreto JB Andreu P Vasquez L Tawfik D Kolhatkar N Coussens LM CD4+ T Cells Regulate Pulmonary Metastasis of Mammary Carcinomas by Enhancing Protumor Properties of Macrophages Cancer Cell 2009 16 91 102 10.1016/j.ccr.2009.06.018 19647220 PMC2778576

[B34] Chang HY Sneddon JB Alizadeh AA Sood R West RB Montgomery K Chi J-T Rijn Mvd Botstein D Brown PO Gene Expression Signature of Fibroblast Serum Response Predicts Human Cancer Progression: Similarities between Tumors and Wounds PLoS Biol 2004 2 e7 10.1371/journal.pbio.0020007 14737219 PMC314300

[B35] Zambelli F Pesole G Pavesi G Pscan: finding over-represented transcription factor binding site motifs in sequences from co-regulated or co-expressed genes Nucl Acids Res 2009 37 W247 252 10.1093/nar/gkp464 19487240 PMC2703934

[B36] Dennis G Sherman B Hosack D Yang J Gao W Lane HC Lempicki R DAVID: Database for Annotation, Visualization, and Integrated Discovery Genome Biology 2003 4 P3 10.1186/gb-2003-4-5-p3 12734009 PMC193660

[B37] Huang DW Sherman BT Lempicki RA Systematic and integrative analysis of large gene lists using DAVID bioinformatics resources Nat Protocols 2008 4 44 57 10.1038/nprot.2008.211 19131956

[B38] Vlieghe D Sandelin A De Bleser PJ Vleminckx K Wasserman WW van Roy F Lenhard B A new generation of JASPAR, the open-access repository for transcription factor binding site profiles Nucl Acids Res 2006 34 D95 97 10.1093/nar/gkj115 16381983 PMC1347477

[B39] Dalton S Treisman R Characterization of SAP-1, a protein recruited by serum response factor to the c-fos serum response element Cell 1992 68 597 612 10.1016/0092-8674(92)90194-H 1339307

[B40] Wang Y Liu D-P Chen P-P Koeffler HP Tong X-J Xie D Involvement of IFN Regulatory Factor (IRF)-1 and IRF-2 in the Formation and Progression of Human Esophageal Cancers Cancer Res 2007 67 2535 2543 10.1158/0008-5472.CAN-06-3530 17363571

[B41] Huang Suyun B CD Van Arsdall Melissa Fidler Isaiah J Stat1 negatively regulates angiogenesis, tumorigenicity and metastasis of tumor cells Oncogene 2002 21 2504 2512 10.1038/sj.onc.1205341 11971185

[B42] Khodarev NN Roach P Pitroda SP Golden DW Bhayani M Shao MY Darga TE Beveridge MG Sood RF Sutton HG STAT1 Pathway Mediates Amplification of Metastatic Potential and Resistance to Therapy PLoS ONE 2009 4 e5821 10.1371/journal.pone.0005821 19503789 PMC2688034

[B43] Woelfle U Assmann V Pantel K Conditionally active STAT1 and its functional role in tumor progression and invasion AACR Meeting Abstracts 2006 2006 430 a-

[B44] Yu H Pardoll D Jove R STATs in cancer inflammation and immunity: a leading role for STAT3 Nat Rev Cancer 2009 9 798 809 10.1038/nrc2734 19851315 PMC4856025

[B45] Richardson AL Wang ZC De Nicolo A Lu X Brown M Miron A Liao X Iglehart JD Livingston DM Ganesan S X chromosomal abnormalities in basal-like human breast cancer Cancer Cell 2006 9 121 132 10.1016/j.ccr.2006.01.013 16473279

[B46] Wilson CL Miller CJ Simpleaffy: a BioConductor package for Affymetrix Quality Control and data analysis Bioinformatics 2005 21 3683 3685 10.1093/bioinformatics/bti605 16076888

[B47] Rhodes DR Yu J Shanker K Deshpande N Varambally R Ghosh D Barrette T Pandey A Chinnaiyan AM ONCOMINE: a cancer microarray database and integrated data-mining platform Neoplasia 2004 6 1 6 15068665 10.1016/s1476-5586(04)80047-2 PMC1635162

[B48] Fisher RA Statistical methods for research workers Edinburgh Oliver and Boyd 1950 354

[B49] Stouffer SA DeVinney LC Suchmen EA The American soldier: Adjustment during army life 1949 Princeton University Press Princeton, NJ

[B50] Hong F Breitling R A comparison of meta-analysis methods for detecting differentially expressed genes in microarray experiments Bioinformatics 2008 24 374 382 10.1093/bioinformatics/btm620 18204063

