# Supplementary figures and images for: Exploring molecular links between lymph node invasion and cancer prognosis in human breast cancer
Source: BMC Syst Biol. 2011 Dec 14;5(Suppl 2):S4. doi: 10.1186/1752-0509-5-S2-S4 (PMC3287484; doi:10.1186/1752-0509-5-S2-S4)

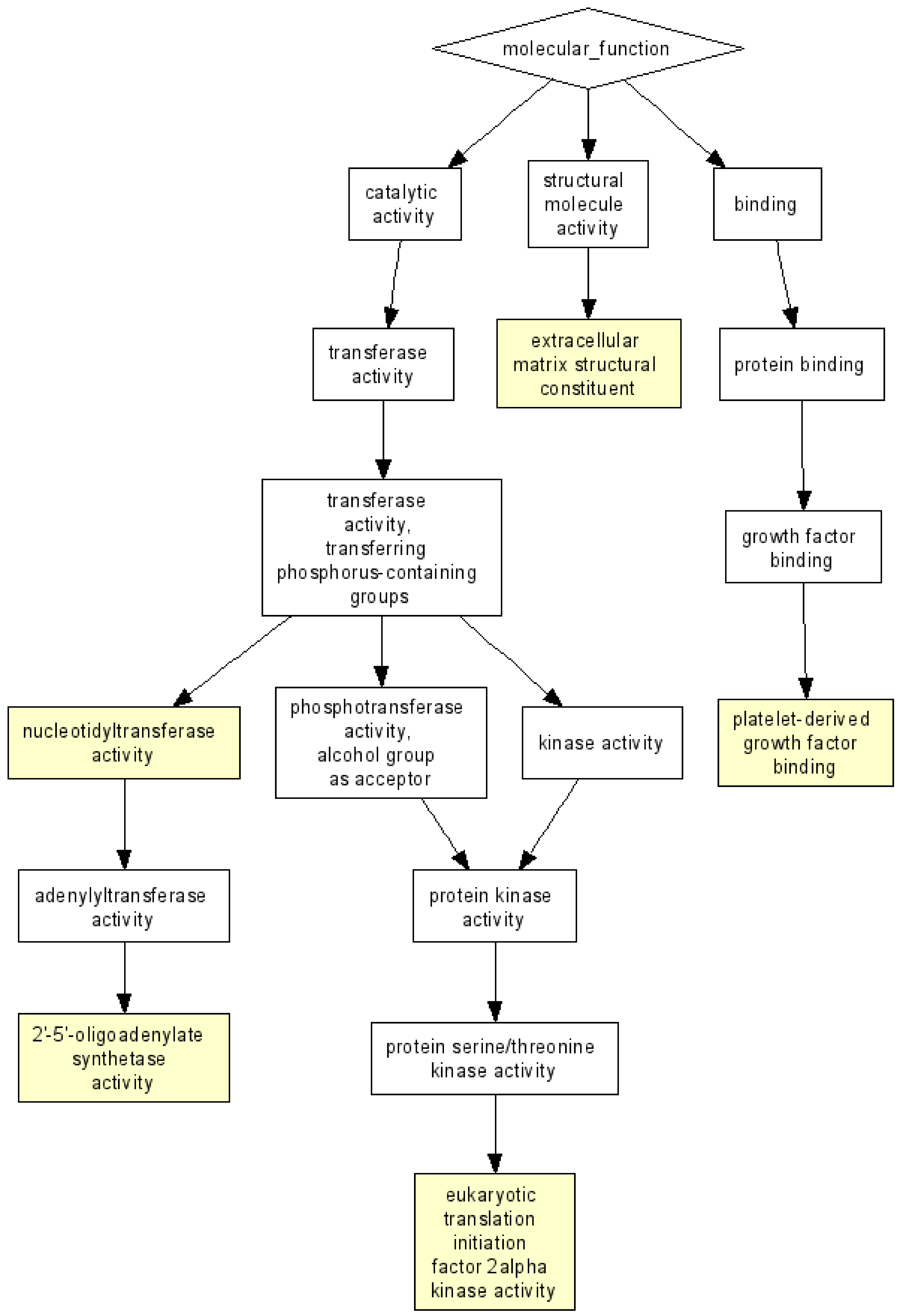

Supplement: Additional File 1 — Analysis of enriched GO Term with GOrilla (N-MEG0→1). Genes related to N0→N1 stage transition showed significant over-representation with collagen and extracellular matrix constituent. [file 1752-0509-5-S2-S4-S1.tif]

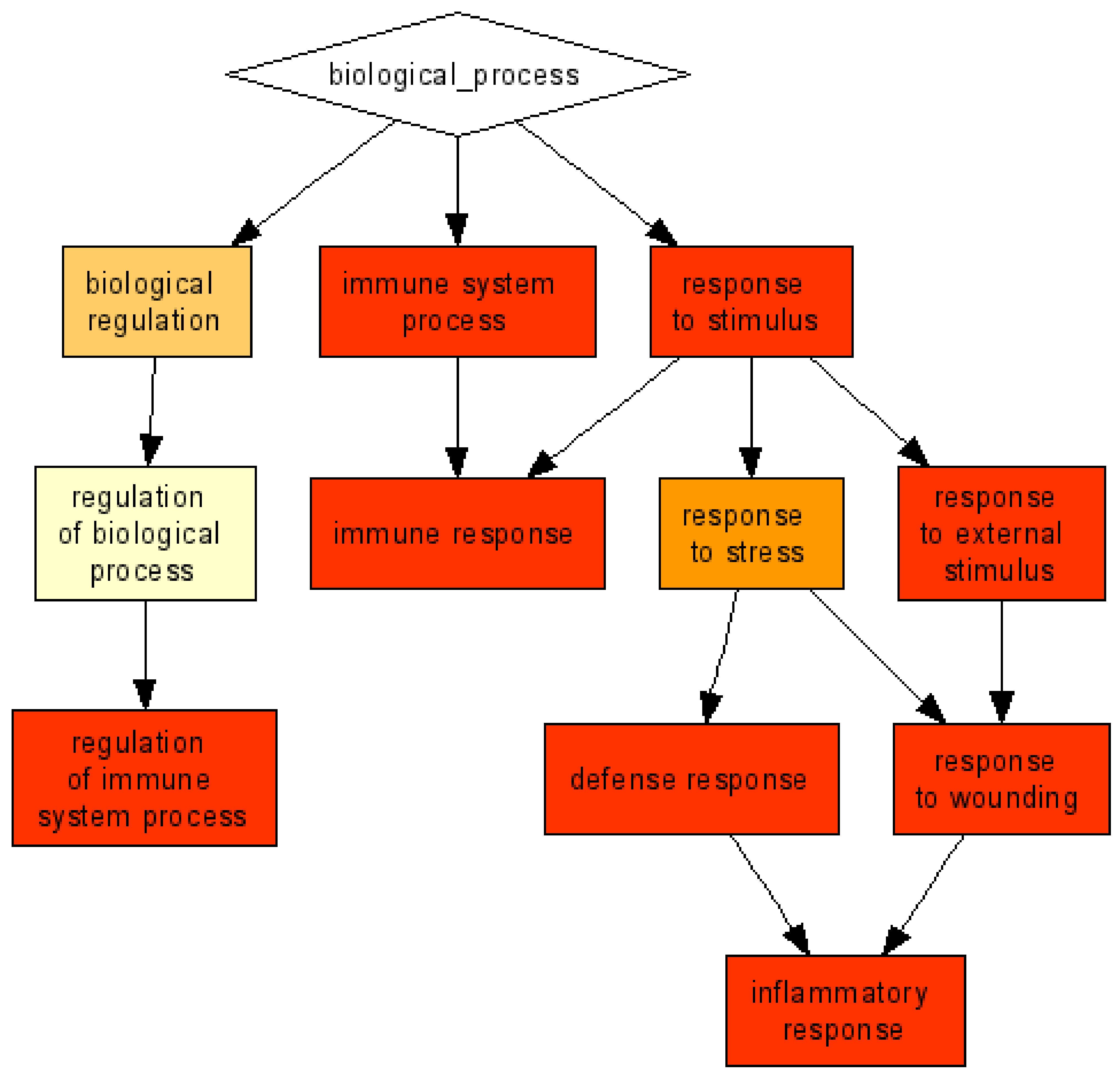

Supplement: Additional File 2 — Analysis of enriched GO Term with GOrilla (N-MEG2→3). Genes related to N2→N3 stage transition showed significant over-representation with immune response, wound response and inflammation. [file 1752-0509-5-S2-S4-S2.tif]
